# Gasping during refractory out-of-hospital cardiac arrest is a prognostic marker for favourable neurological outcome following extracorporeal cardiopulmonary resuscitation: a retrospective study

**DOI:** 10.1186/s13613-020-00730-3

**Published:** 2020-08-10

**Authors:** Naofumi Bunya, Hirofumi Ohnishi, Kenshiro Wada, Ryuichiro Kakizaki, Takehiko Kasai, Nobutaka Nagano, Nobuaki Kokubu, Kei Miyata, Shuji Uemura, Keisuke Harada, Eichi Narimatsu

**Affiliations:** 1 Department of Emergency Medicine, Sapporo Medical University, S1W16 Chuo-ku Sapporo, Hokkaido, 060-8543 Japan; 2 Department of Public Health, Sapporo Medical University, Sapporo, Japan; 3 Department of Cardiovascular, Renal and Metabolic Medicine, Sapporo Medical University, Sapporo, Japan; 4 Department of Neurosurgery, Sapporo Medical University, Sapporo, Japan

**Keywords:** Gasping, Extracorporeal cardiopulmonary resuscitation, Extracorporeal membrane oxygenation, Out-of-hospital cardiac arrest, Agonal respiration

## Abstract

**Background:**

Gasping during cardiac arrest is associated with favourable neurological outcomes for out-of-hospital cardiac arrest. Moreover, while extracorporeal cardiopulmonary resuscitation (ECPR) performed for refractory cardiac arrest can improve outcomes, factors for favourable neurological outcomes remain unknown. This study aimed to examine whether gasping during cardiac arrest resuscitation during transport by emergency medical services (EMS) was independently associated with a favourable neurological outcome for patients who underwent ECPR. This retrospective study was based on medical records of all adult patients who underwent ECPR due to refractory cardiac arrest. The primary endpoint was neurologically intact survival at discharge. The study was undertaken at Sapporo Medical University Hospital, a tertiary care centre approved by the Ministry of Health, Labour and Welfare, located in the city of Sapporo, Japan, between January 2012 and December 2018.

**Results:**

Overall, 166 patients who underwent ECPR were included. During transportation by EMS, 38 patients exhibited gasping, and 128 patients did not. Twenty patients who exhibited gasping during EMS transportation achieved a favourable neurological outcome (20/38; 52.6%); 14 patients who did not exhibit gasping achieved a favourable neurological outcome (14/128; 10.9%). Gasping during transportation by EMS was independently associated with favourable neurological outcome irrespective of the type of analysis performed (multiple logistic regression analysis, odds ratio [OR] 9.52; inverse probability of treatment weighting using propensity score, OR 9.14).

**Conclusions:**

The presence of gasping during transportation by EMS was independently associated with a favourable neurological outcome in patients who underwent ECPR. The association of gasping with a favourable neurological outcome in patients with refractory cardiac arrest suggests that ECPR may be considered in such patients.

## Background

Out-of-hospital cardiac arrest (OHCA) is a major public health issue in developed countries, with approximately 120,000 OHCA events occurring in Japan annually. Patient survival after OHCA remains low [1, 2]. Moreover, among survivors, only 3–10% achieve a favourable neurological outcome [2–5], likely due to insufficient cerebral blood flow. Although minimised interruption of chest compressions has been emphasised to maintain the blood flow, chest compressions alone are inadequate to ensure sufficient cerebral blood flow [6]; accordingly, prolonged cardiopulmonary resuscitation (CPR) without the return of spontaneous circulation (ROSC) results in an unfavourable outcome following cardiac arrest. To restore cerebral blood flow, resuscitation using extracorporeal membrane oxygenation (ECMO) was proposed in the 1960s [7, 8]. Several recent studies have suggested that better neurological outcomes are achieved after refractory cardiac arrest in patients who receive extracorporeal cardiopulmonary resuscitation (ECPR) [9–11].

ECPR is a recently adopted approach supported by many studies for refractory cardiac arrest; however, established inclusion criteria remain unclear. Some characteristics have been associated with good neurological outcomes in the setting of ECPR; these include presence of an initial shockable cardiac rhythm (ventricular fibrillation [VF] and pulseless ventricular tachycardia [VT]), witnessed cardiac arrest, presence of bystander CPR attempt, shorter duration of no-flow and low-flow time, cardiac aetiology, higher arterial pH, and initial lower serum lactate concentration [12–17]. Despite these known variables, only 11–15% of patients achieve a favourable neurological outcome using ECPR following an OHCA [10, 18]. One indicator of a favourable neurological outcome after OHCA is gasping during cardiac arrest [19]. Furthermore, gasping is associated with witnessed events and VF [20]. These findings suggest that gasping during cardiac arrest may represent an early phase of the arrest.

However, it is still unknown whether gasping is a neurological prognostic factor for refractory cardiac arrest patients who fail to achieve ROSC by the usual resuscitation methods. The primary aim of this study was to examine whether gasping by the patient during transportation by emergency medical services (EMS) would be independently associated with a favourable neurological outcome for patients undergoing ECPR.

## Methods

The present study conformed to the principles outlined in the Declaration of Helsinki and was performed with the approval of the institutional ethical committee of Sapporo Medical University (Number: 312-1118). Informed consent requirement was waived because of the life-threatening situation and the absence of a therapeutic alternative. Information was delivered to the patients’ relatives after inclusion, as appropriate, in a life-threatening context.

### Setting

The study was undertaken at Sapporo Medical University Hospital, a tertiary care centre approved by the Ministry of Health, Labour and Welfare and located in the city of Sapporo, Japan. Sapporo City covers 1121 km^2^ of land area and has a population of approximately 1.9 million. Between January 2012 and December 2018, we retrospectively analysed consecutive patients who met all the following inclusion criteria: OHCA occurrence, age  ≥ 18 years, admission to our institution and resuscitation with ECPR.

### ECMO management and post-resuscitation care

The decision to initiate ECPR was dependent on the attending physician who treated the patient upon arrival. ECMO was implemented in the emergency department by the ECPR team, which comprised well-trained emergency physicians, cardiologists, and clinical engineers. In general, ECPR was initiated if ROSC did not occur or could not be maintained during transportation and if the patient was assessed to have good activities of daily life before cardiac arrest based on an interview with the patient’s relatives. Emergency physicians and cardiologists tended to initiate ECPR in nonelderly patients with refractory VF, witnessed cardiac arrest, and/or presence of bystander CPR attempt, based on experience.

### Data collection and assessment of neurological outcome

The primary endpoint was survival with favourable neurological function, evaluated using the cerebral performance category (CPC) on the day of hospital discharge. A CPC score of 1–2 was regarded as favourable, while a CPC score of 3–5 was regarded as unfavourable. We compared the baseline characteristics and pre-hospital clinical data, including gasping during transportation by EMS and cardiac arrest causes, between the favourable and unfavourable outcome groups. There is an established form used for the medical records of ECPR patients in our facility. A blank field in the form has to be completed with respect to gasping during transportation. It is necessary for the attending physicians to ensure that this blank field is completed when the ECPR patient is admitted to our facility. No-flow time was defined as the time from collapse to initiation of CPR, while low-flow time was defined as the time from CPR initiation to ECMO active running.

### Statistical analysis

Continuous variables are presented herein as median with interquartile ranges; such variables were compared using the Mann–Whitney *U* test. Categorical variables were compared using Fisher’s exact test. A multivariate logistic regression analysis was performed to evaluate the association between gasping and ECPR. For the multivariate logistic regression analysis, the initial cardiac rhythm, low-flow time, and serum lactate value were selected as populated variables with reference to previous reports [13, 21], and gasping during transportation, which is the subject of this study, was also added. Additionally, to adjust for potential baseline differences between the presence and absence of gasping during EMS transportation groups, we adopted propensity score (PS) analysis and generated PS concerning the patients’ likelihood of gasping during EMS transportation. The PS model was estimated using a logistic regression model that adjusted for age, sex, cardiac arrest aetiology of cardiac origin, witness of collapse, bystander CPR attempt, initial cardiac rhythm, cardiac rhythm on admission to the resuscitation bay, low-flow time, adrenaline administration during transportation, and adrenaline administration at the resuscitation bay. The c-statistic for the PS model was 0.77. We applied inverse probability treatment weighting (IPTW) by PS. Two-sided tests were used, and *p* values less than 0.05 were considered statistically significant. All statistical analyses were performed using SPSS Statistics version 25 (IBM Corp, Armonk, NY, US) and EZR (Saitama Medical Centre, Jichi Medical University, Saitama, Japan), which is a graphical user interface for R (The R foundation for Statistical Computing, Vienna, Austria).

## Results

**Fig. 1:**
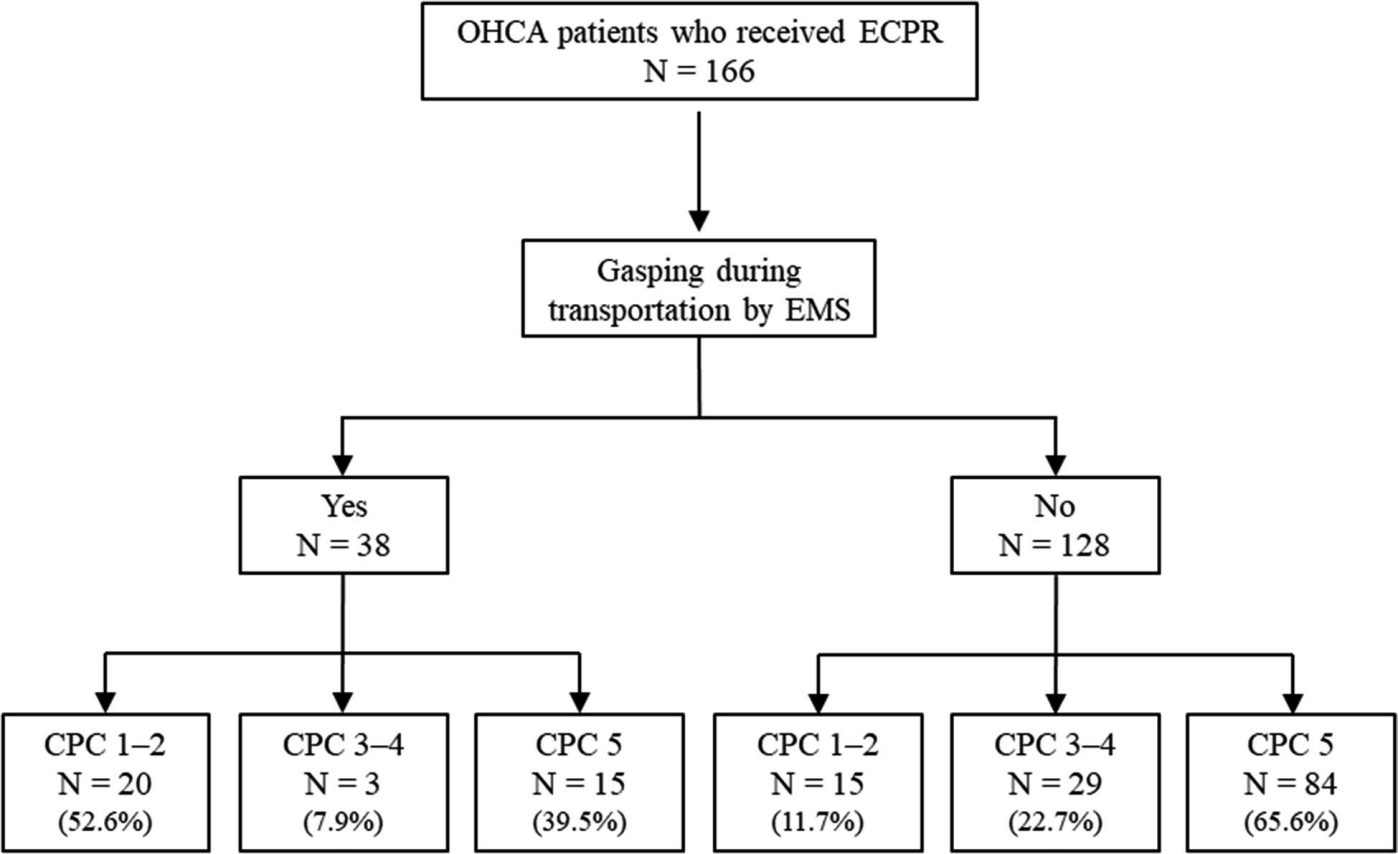
Patient outcomes based on patient gasping during transportation by emergency medical services. *EMS* emergency medical services, *OHCA* out-of-hospital cardiac arrest, *ECPR* extracorporeal cardiopulmonary resuscitation, *CPC* cerebral performance category

A total of 166 ECPR patients were included in the study. During transportation by EMS, 38 patients exhibited gasping, and 128 patients did not exhibit gasping (Fig. 1). Table 1 shows the patients’ characteristics based on the neurological outcome. Thirty-five patients had a favourable neurological outcome, and 131 patients had an unfavourable neurological outcome. Among patients who achieved a favourable neurological outcome, 20 exhibited gasping during EMS transportation (20/35; 57.1%). The patients in the favourable neurological outcome group were younger than those in the unfavourable neurological outcome group (Table 1). Compared with the unfavourable neurological outcome group, the favourable neurological outcome group had a higher rate of bystander CPR attempt, gasping during transportation, and achieving ROSC at admission. Compared with the unfavourable neurological outcome group, the favourable neurological outcome group had a lower dosage of adrenaline administered both during EMS transportation and in hospital, as well as a shorter duration of no-flow time. The sensitivity, specificity, positive predictive value, and negative predictive value of gasping for achieving a favourable neurological outcome were 57.1%, 86.3%, 52.6%, and 88.3%, respectively. Table 2 shows the results of the logistic regression analysis according to neurological outcome. Multivariate logistic regression analysis showed that gasping during transportation by EMS was independently associated with a favourable neurological outcome (odds ratio [OR]: 9.94; 95% confidence interval [CI] 5.49–18.00) after adjusting for initial cardiac rhythm, low-flow time, and serum lactate value.

**Table 1:** Comparison of baseline characteristics based on neurological outcome

variables	Favourable neurological outcome, *n* = 35	Unfavourable neurological outcome, *n* = 131	*P* value
Age (y)	53.0 (43.0, 64.5)	63.0 (50.0, 70.0)	0.004
Male sex, *n* (%)	26 (74.3)	102 (77.9)	0.655
Cardiac arrest witnessed, *n* (%)	24 (68.6)	88 (67.2)	0.876
Bystander CPR attempt, *n* (%)	25(71.4)	63(48.1)	0.014
Initial recorded cardiac arrest rhythm, *n* (%)			0.709
VF or pulseless VT	22 (62.9)	89 (67.9)	
Pulseless electrical activity	7 (20.0)	23 (17.6)	
Asystole	6 (17.1)	19 (14.5)	
Gasping during transportation by EMS, *n* (%)	20 (57.1)	18 (13.7)	< 0.001
Aetiology of cardiac arrest, *n* (%)			0.222
Cardiac, *n* (%)	25 (71.4)	106 (80.9)	
No cardiac, *n* (%)	10 (28.6)	25 (19.1)	
Pulmonary embolism	1	2	
Subarachnoid haemorrhage	0	3	
Accidental hypothermia	8	6	
Acute aortic dissection	0	3	
Respiratory disease			
Pneumonia	0	1	
Chronic obstructive pulmonary disease	0	1	
Asthma	0	1	
Other	1	8	
Epinephrine dose before hospital admission, mg	0.0 (0.0, 2.0)	2.0 (0.0, 3.0)	0.002
Cardiac rhythm at admission			0.009
ROSC^a^	6 (17.1)	4 (3.1)	
VF or pulseless VT	19 (54.3)	59 (45.0)	
Pulseless electrical activity	6 (17.1)	46 (35.1)	
Asystole	4 (11.4)	22 (16.8)	
Low-flow duration, min	42.00 (30.00, 55.00)	46.00 (36.00, 54.00)	0.299
No-flow duration, min, median, *n* = 115^b^	0.00 (0.00, 1.50), *n* = 26^b^	2.00 (0.00, 10.00), *n* = 89^b^	0.006
pH at admission, *n* = 162^c^	6.94 (6.88, 7.06), *n* = 35	6.90 (6.81, 7.02), *n* = 127	0.098
Serum lactate at admission, *n* = 158^c^	13.7 (8.7, 16.0), *n* = 35	12.7 (11.0, 15.2), *n* = 123	0.948
Epinephrine administration after admission, mg	1.0 (0.0, 2.0)	1.0 (0.0, 3.0)	0.036
Intra-aortic balloon pumping, *n* (%)	26 (74.3)	89 (67.9)	0.470
Percutaneous coronary intervention, *n* (%)	16 (45.7)	62 (47.3)	0.865
Therapeutic temperature management, *n* (%)	26 (74.3)	84 (64.1)	0.259

Numbers shown in parentheses are percentages, and continuous variables were presented as median with interquartile ranges

*CPR* cardiopulmonary resuscitation, *VF* ventricular fibrillation, *VT* ventricular tachycardia, *EMS* emergency medical service, *ROSC* return of spontaneous circulation

^a^These cases exhibited repeated ROSC and cardiac arrest. Therefore, these patients underwent extracorporeal cardiopulmonary resuscitation

^b^No-flow time could be basically calculated in patients with witnessed cardiac arrest while that for some un-witnessed cardiac arrest patients, no-flow time could be estimated by relatives and/or bystanders. Accordingly, the number of patients with no-flow was less than 166

^c^The numbers were less than 166 due to insufficient data

**Table 2:** Logistic regression analysis for favourable neurological outcome

Variables	Unadjusted odds ratio (95% CI) n = 166	*P* value	Adjusted odds ratio^A^ (95% CI) n = 166^a^	*P* value	Adjusted odds ratio^B^ (95% CI) n = 158^a^	*P* value
Age (y)	0.96 (0.93–0.99)	0.003				
Male sex	0.82 (0.35–1.95)	0.655				
Cardiac arrest witnessed	1.07 (0.48–2.38)	0.876				
Bystander CPR attempt	2.70 (1.20–6.06)	0.016				
Initial recorded cardiac arrest rhythm			0.62 (0.42–0.93)	0.02	0.63 (0.42–0.95)	0.028
VF or pulseless VT	0.78 (0.28–2.19)	0.641				
Pulseless electrical activity	0.96 (0.28–3.36)	0.954				
Asystole	1.000 (ref.)	0.850				
Gasping during transportation by EMS	8.37 (3.64–19.27)	<.001	9.52 (5.31–17.08)	< .001	9.94 (5.49–18.00)	< .001
Aetiology of cardiac arrest	0.59 (0.25–1.38)	0.225				
Epinephrine dose before hospital admission, mg	0.70 (0.54–0.92)	0.009				
Cardiac rhythm at admission						
ROSC	8.25 (1.58–43.13)	0.012				
VF or pulseless VT	1.77 (0.54–5.79)	0.344				
Pulseless electrical activity	0.72 (0.18–2.80)	0.633				
Asystole	1.000 (ref.)	0.013				
Low-flow duration, min	0.98 (0.95–1.00)	0.075	0.99 (0.97–1.01)	0.34	0.99 (0.97–1.01)	0.510
No-flow duration, min, n = 115^b^	0.88 (0.80–0.98)	0.018				
pH at admission, n = 162^c^	9.88 (0.77–127.38)	0.079				
Serum lactate value at admission, n = 158^c^	1.01 (0.93–1.10)	0.783			1.03 (0.97–1.09)	0.399
Epinephrine administration after admission, mg	0.76 (0.57–1.02)	0.070				
Intra-aortic balloon pumping	1.36 (0.59–3.17)	0.471				
Percutaneous coronary intervention	0.94 (0.44–1.98)	0.865				
Therapeutic temperature management	1.62 (0.70–3.74)	0.261				

*CPR* cardiopulmonary resuscitation, *VF* ventricular fibrillation, *VT* ventricular tachycardia, *EMS* emergency medical service, *ROSC* return of spontaneous circulation

^a^For the multivariate logistic analysis, the initial cardiac rhythm, and low-flow time were selected as populated variables with reference to previous reports [13, 21], and gasping during transportation, which is the subject of this study, were also added (showing adjusted odds ratio^A^). In the adjusted odds ratio^B^, the serum lactate value for which the sample number missing was also entered in the multivariate logistic analysis

^b^No-flow time could be basically calculated in patients with witnessed cardiac arrest, while for some un-witnessed cardiac arrest patients, no-flow time could be estimated by relatives and/or bystanders. Accordingly, the number of patients with no-flow time was less than 166

^c^The number was less than 166 due to insufficient data

### Gasping-related variables

Patients who demonstrated gasping (Gasping group) during transportation by EMS were more likely to have received bystander CPR than were patients who did not demonstrate gasping (Non-gasping group) (73.7% vs 46.9%, *p* = 0.005). Moreover, the Gasping group was more likely to have had a cardiac arrest witness than was the Non-gasping group (89.5% vs 60.9%, *p* = 0.001) (Table 3). In both groups, VF/VT was the most frequent initial cardiac rhythm. Furthermore, the Gasping group had a shorter no-flow time (median, 0 min vs 6 min, *p* = 0.002) and low-flow time (40.5 min vs 46.0 min, *p* = 0.049) compared to those of the Non-gasping group. Twenty patients (52.6%) in the Gasping group exhibited a favourable neurological outcome compared with 14 patients (10.9%) in the Non-gasping group (unadjusted OR: 9.05; 95% CI 3.89–21.06; *p* < 0.001) (Table 3). IPTW was used to adjust the background, and the standardized differences below 0.1 included age, sex, bystander CPR attempt, cardia arrest aetiology, cardiac rhythm at admission, VF/VT, no-flow time, serum lactate value, epinephrine administration from EMS, and epinephrine administration after admission (Table 3). The likelihood of a favourable neurological outcome at discharge in the Gasping group was significantly higher than that in the Non-gasping group (OR: 9.14; 95% CI 3.36–24.85; *p* < 0.001) (Table 4).

**Table 3:** Comparison of baseline characteristics according to existence of gasping during transportation by EMS

Variables	Univariate analysis				IPTW
	Gasping during transportation n = 38	No gasping during transportation n = 128	*P* value	Std Dif	Std Dif
Age (y)	61.0 (52.0, 70.8)	59.5 (47.3, 69.0)	0.435	0.172	0.052
Male sex, *n* (%)	28 (73.7)	100 (78.1)	0.660	0.104	0.034
Cardiac arrest witnessed, *n* (%)	34 (89.5)	78 (60.9)	0.001	0.700	0.341
Bystander CPR attempt, *n* (%)	28 (73.7)	60 (46.9)	0.005	0.570	0.095
Initial recorded cardiac arrest rhythm, *n* (%)			0.002		
VF or pulseless VT	22 (57.9)	89 (69.5)		0.244	0.363
Pulseless electrical activity	14 (36.8)	16 (12.5)		0.589	0.492
Asystole	2 (5.3)	23 (18.0)		–	–
Aetiology of CA, *n* (%)			1.000		
Cardiac, *n* (%)	30 (78.9)	101 (78.9)		0.001	0.014
No cardiac, *n* (%)	8 (21.1)	27 (21.1)			
Pulmonary embolism	1	2			
Subarachnoid haemorrhage	0	3			
Accidental hypothermia	3	11			
Acute aortic dissection	1	2			
Respiratory disease					
Pneumonia	0	1			
Chronic obstructive pulmonary disease	1	0			
Asthma	1	0			
Other	1	8			
Epinephrine administration from EMS, mg	1.0 (0.0, 2.0)	2.0 (0.0, 3.0)	0.025	0.443	0.083
Cardiac rhythm at admission			0.009		
ROSC	5 (13.2)	5 (3.9)		0.336	0.016
VF or pulseless VT	17 (44.7)	61 (47.7)		0.059	0.198
Pulseless electrical activity	15 (39.5)	37 (28.9)		0.224	0.544
Asystole	1 (2.6)	25 (19.5)		–	–
Low-flow duration, min	40.5 (29.5, 51.3)	46.0 (37.0, 54.0)	0.049	0.397	0.130
No-flow duration, min^a^	0.0 (0.0, 5.0), n = 35^b^	6.0 (0.0, 10.0), n = 82^b^	0.002	0.586	0.012
pH at admission, n = 162^b^	6.914 (6.814, 7.012), n = 37	6.914 (6.815, 7.019), n = 125	0.795	0.104	0.128
Serum lactate at admission, n = 158^b^	13.8 (9.5, 16.0), n = 36	12.7 (10.8, 15.1), n = 122	0.435	0.217	0.036
Epinephrine administration after admission, mg	1.0 (0.0, 3.0)	1.0 (0.0, 2.0)	0.841	0.029	0.054
Intra-aortic balloon pumping, *n* (%)	29 (76.3)	86 (67.7)	0.421	0.204	0.138
Percutaneous coronary intervention, *n* (%)	16 (42.1)	62 (48.8)	0.579	0.127	0.280
Therapeutic temperature management, *n* (%)	26 (68.4)	84 (66.1)	0.847	0.060	0.192
Outcomes					
Survival discharge, *n* (%)	23 (60.5)	44 (34.4)	0.005		
CPC score 1–2 at hospital discharge, *n* (%)	20 (52.6)	14 (10.9)	< 0.001		

*IPTW* inverse probability treatment weighting, *EMS* emergency medical service, *CPR* cardiopulmonary resuscitation, *VF* ventricular fibrillation, *VT* ventricular tachycardia, *ROSC* return of spontaneous circulation, *CPC* cerebral performance category, *Std Dif* standardised difference

^a^No-flow time could be basically calculated in patients with cardiac arrest witnessed and some un-witnessed cardiac arrest patients could be estimated no-flow time by relatives and/or bystanders. Accordingly, the number of patients with no-flow was less than 166

_b_The number was less than 166 due to insufficient data

**Table 4:** Analyses of gasping during transportation by EMS effect on favourable neurological outcome

Analysis method	Odds ratio	95% CI	*P* value
Unadjusted	8.37	3.64–19.27	< .001
Adjusted using logistic regression	9.52	5.31–17.08	< .001
IPTW using PS	9.14	3.36–24.85	< .001

The IPTW method creates a synthetic sample in which the distribution of measured baseline covariates is independent of exposure assignment. The weight of each participant is calculated using two variables: the participant’s exposure status (0 for absence of gasping and 1 for presence of gasping) and the PS of the participant. The weight of the participant is equal to the inverse of the probability of receiving the treatment that the participant actually received

*EMS* Emergency Medical Services, *PS* propensity score, *IPTW* Inverse probability of treatment weighting, *CI* confidence interval

## Discussion

The present study shows that gasping in patients who experienced OHCA during EMS transportation was independently associated with favourable neurological outcome in patients who underwent ECPR. The underlying reason for the relationship between gasping and favourable neurological outcome was suggested by Debaty et al., who remarked that gasping may be an important natural biomarker for the presence of the brainstem activity during CPR [19]. Accordingly, gasping during CPR directly and partially reflects the brainstem activity. In contrast, other reported prognostic factors do not directly reflect the brain activity [12–17].

In the present study, bystander CPR and shorter duration of no-flow time were also associated with the presence of gasping during EMS transportation. A previous study suggested that in the absence of adequate CPR, gasping was short-lived and rapidly declined [22]. Preserved gasping is considered to result from at least some level of maintenance of cerebral blood flow due to chest compressions. Thus, preserved gasping may indirectly suggest that there was only a short duration during which no adequate CPR was administered. Therefore, gasping directly indicates that the brain is partially functioning and indirectly indicates a short duration without adequate CPR. This may explain the association between preserved gasping and a favourable neurological outcome in patients after ECPR.

Additionally, gasping by the patient during EMS transportation is a prognostic factor for favourable neurological outcome; therefore, this important information can be used to prepare ECPR for refractory cardiac arrest. Accordingly, during transportation to the hospital, we propose that EMS had better methods for transmitting information regarding the patients gasping during cardiac arrest. As previously indicated, there is no properly consolidated system regarding the recording, tracking, and transfer of information such as the existence of gasping during cardiac arrest [19]. Therefore, we think that it is better to include information regarding gasping in a manner that reliably delivers such information, similar to that used for initial cardiac rhythms, cardiac arrest witnessed, and the presence of bystander CPR. Such information may benefit potential patients owing to ECPR implementation. We recommend that hospitals that receive patients in refractory cardiac arrest establish such a system to prepare and better utilise ECPR.

Low-flow time, which has been associated with prognosis in many studies [14, 17], was not associated with neurological outcomes in this study. It has been shown that a shorter low-flow time increases the likelihood of favourable neurological outcomes, and awareness of resuscitation time is certainly important for EMS personnel and emergency physicians. In our facility, since we regard the presence of gasping to be associated with favourable neurological outcomes, when determining whether to use ECPR, the low-flow time may have been underestimated in relation to the presence of gasping. Therefore, this may have resulted in a biased conclusion that low-flow time was not associated with neurological outcomes in this study. Awareness of the significance of both gasping and low-flow time is important for resuscitation.

There were several limitations to this study. First, this was a single-centre, retrospective study with a relatively small sample size, which might have led to statistical error by allowing unrecognised bias. In particular, the small sample size limited the number of covariates that could be entered into the multivariate analysis. In addition, although we used IPTW by PS, since gasping is associated with other variables (such as no flow time) as well as the outcome, the current statistical analysis is insufficient to fully demonstrate the implications of gasping. Additionally, as the standardized differences of some variables were not below 0.1, the result of the IPTW was also insufficient from this point of view. Therefore, the purpose of the IPTW was to support the results of multivariate analysis. Further analysis that considers other confounding factors is necessary. Second, we used data only from patients who underwent ECPR. Although our facility has an ECPR database, it does not cover the entire OHCA. For this reason, the frequency of patients who maintained gasping but in whom ECPR was not introduced was unknown. This is a major limitation in recommending ECPR for refractory cardiac arrest with gasping. For instance, some patients who did not undergo ECPR might have been suitable candidates for ECPR. Furthermore, we were unable to assess whether patients who had preserved gasping during resuscitation but who did not undergo ECPR could have achieved a favourable neurological outcome by undergoing ECPR. Since the frequency of cardiac arrest patients who maintained gasping but in whom ECPR was not introduced was unknown, the algorithm for introducing ECPR by the presence or absence of gasping could not be reported. Third, we evaluated the outcomes at the time of hospital discharge, and long-term outcomes were not evaluated. Fourth, depending on the attending physician, ECPR was often introduced if gasping was preserved. We have experienced several ECPR cases with a favourable neurological outcome in patients with gasping preserved during cardiac arrest. Based on the experiences, for example, ECPR was sometimes introduced in cases, where gasping was preserved, even if the initial cardiac rhythm was non-shockable or low-flow time was quite long. This may be a selection bias in the study.

## Conclusions

The present study shows that gasping by the patient during transportation by EMS was independently associated with a favourable neurological outcome in patients undergoing ECPR. Since gasping has the potential to confer a favourable neurological outcome in patients experiencing refractory cardiac arrest, it is important to raise awareness of the potential importance of gasping by informing EMS personnel and emergency physicians on the positive implications of gasping so that they do not abandon resuscitation efforts in such instances. In these cases, the use of ECPR can be considered.

## Data Availability

The datasets used and/or analysed during the current study are available from the corresponding author on reasonable request.

## Abbreviations

CI: Confidence interval
CPC: Cerebral performance category
CPR: Cardiopulmonary resuscitation
ECMO: Extracorporeal membrane oxygenation
ECPR: Extracorporeal cardiopulmonary resuscitation
EMS: Emergency medical services
IPTW: Inverse probability treatment weighting
OHCA: Out-of-hospital cardiac arrest
OR: Odds ratio
PS: Propensity score
ROSC: Return of spontaneous circulation
VF: Ventricular fibrillation
VT: Ventricular tachycardia
